# Antimelanogenic effect of c-phycocyanin through modulation of tyrosinase expression by upregulation of ERK and downregulation of p38 MAPK signaling pathways

**DOI:** 10.1186/1423-0127-18-74

**Published:** 2011-10-11

**Authors:** Li-Chen Wu, Yu-Yun Lin, Szu-Yen Yang, Yu-Ting Weng, Yi-Ting Tsai

**Affiliations:** 1 Department of Applied Chemistry, National Chi Nan University, Puli, Nantou, 545, Taiwan; 2 Graduate Institute of Biomedicine and Biomedical technology, National Chi Nan University, Puli, Nantou, 545, Taiwan

**Keywords:** C-phycocyanin, antimelanogenesis, CREB, MITF, MAPK/ERK, p38 MAPK

## Abstract

**Background:**

Pigmentation is one of the essential defense mechanisms against oxidative stress or UV irradiation; however, abnormal hyperpigmentation in human skin may pose a serious aesthetic problem. C-phycocyanin (Cpc) is a phycobiliprotein from spirulina and functions as an antioxidant and a light harvesting protein. Though it is known that spirulina has been used to reduce hyperpigmentation, little literature addresses the antimelanogenic mechanism of Cpc. Herein, we investigated the rationale for the Cpc-induced inhibitory mechanism on melanin synthesis in B16F10 melanoma cells.

**Methods:**

Cpc-induced inhibitory effects on melanin synthesis and tyrosinase expression were evaluated. The activity of MAPK pathways-associated molecules such as MAPK/ERK and p38 MAPK, were also examined to explore Cpc-induced antimelanogenic mechanisms. Additionally, the intracellular localization of Cpc was investigated by confocal microscopic analysis to observe the migration of Cpc.

**Results:**

Cpc significantly (P < 0.05) reduced both tyrosinase activity and melanin production in a dose-dependent manner. This phycobiliprotein elevated the abundance of intracellular cAMP leading to the promotion of downstream ERK1/2 phosphorylation and the subsequent MITF (the transcription factor of tyrosinase) degradation. Further, Cpc also suppressed the activation of p38 causing the consequent disturbed activation of CREB (the transcription factor of MITF). As a result, Cpc negatively regulated tyrosinase gene expression resulting in the suppression of melanin synthesis. Moreover, the entry of Cpc into B16F10 cells was revealed by confocal immunofluorescence localization and immunoblot analysis.

**Conclusions:**

Cpc exerted dual antimelanogenic mechanisms by upregulation of MAPK/ERK-dependent degradation of MITF and downregulation of p38 MAPK-regulated CREB activation to modulate melanin formation. Cpc may have potential applications in biomedicine, food, and cosmetic industries.

## Background

C-phycocyanin (Cpc), a major type of phycocyanin of phycobilisome in spirulina, has been suggested to exhibit radical-scavenging property [1] to reduce inflammatory responses [2,3] and oxidative stress [1,4]. This phycobiliprotein also induces HeLa cell apoptosis [5,6] enhances wound healing [7], retards platelet aggregation [8,9] and acts as a photodynamic agent to eradicate cancer cells in vitro [10,11]. Moreover, animal studies revealed that Cpc possesses protective effects on tetrachloride-induced hepatocyte damage [12] and oxalate-resulted nephronal impartment [13], and oral administration of Cpc successfully relieves the pathogenicity of activated brain microglia in neurodegenerative disorders [14] and exhibits a preventative effect on viral infection [15].

Recently it is suggested that Cpc regulates the mitogen-activated protein kinases (MAPK) pathways, such as p38 MAPK, and extracellular signal-regulated protein kinases (ERKs). These signaling are known to respond to extracellular stress stimuli to regulate several cellular activities including proliferation, survival/apoptosis, gene expression, and differentiation. Cpc attenuates ischemia/reperfusion (I/R) induced cardiac dysfunction through its antioxidative capacity, antiapoptotic property, suppression of p38 MAPK, and promotion of cardioprotective ERK signaling [16]. The exalted phosphorylation of ERK activates the transcription factors such as c-myc and c-fos. However, this phosphorylation may also lead to the degradation of microphthalmia-associated transcription factor (MITF), a transcription factor associated with cell development, survival and certain activities. Significant degradation of MITF is reported to be phosphorylated at serine 73 (S73) by ERK, leading to subsequent ubiquitin-dependent proteasomal degradation [17]. MITF is critical in transcriptional activation of genes required for melanogenesis (tyrosinase, TYRP1, and TYRP2), survival, as well as the differentiation of melanocytes [18].

The process of melanogenesis constitutes a complex series of enzymatic and chemical reactions. Tyrosinase, a dinuclear type-3 copper-containing mixed function oxidase, initiates melanogenesis through catalyzing the synthesis of melanin by hydroxylation of a monophenol and the subsequent oxidation of o-diphenols into o-quinones. The biosynthesis of this rate-limiting enzyme in melanogenesis is modulated by cell-signaling mechanisms such as PKC-associated pathway and PKA-independent cAMP-dependent Ras pathway (cAMP/Ras/ERK) [19,20]. The upregulation of cAMP is reportedly to activate MAPK/ERK in B16F10 melanoma cells and in normal melanocytes [21]. As Cpc has been linked to regulation of the MAPK/ERK pathway, it would be very likely that Cpc could modulate melanogenesis through cell signaling regulation in addition to its antioxidative capacity.

In the present study, we evaluated the potential of Cpc to be used as an antimelanogenic agent and explored the involvement of ERK and p38 MAPK in Cpc-induced antimelanogenic regulation in B16F10 melanoma cells. To the best of our knowledge, this is the first report addressing the antimelanogenic mechanism of Cpc. The expression of tyrosinase and the production of melanin were determined to examine the antimelanogenic effect of Cpc. The levels of signaling molecules such as cAMP, ERK, p38 MAPK, MITF and CREB were also investigated to delineate the cellular regulatory pathways. Results indicated that Cpc significantly elevated the abundance of cAMP and activated ERK1/2, which promoted the degradation of MITF, leading to the suppression of melanogenesis. Moreover, Cpc attenuated the activation of p38 MAPK and the downstream phosphorylation of CREB to down-regulate the pigmentation. Our data may provide potential applications of Cpc in food industry for antioxidation and anti-browning, in biomedicine industry for abnormal hyperpigmentation, as well as in cosmetics for skin whitening.

## Methods

### Cell line and Cell culture

B16F10 murine melanoma cells (BCRC60031) were purchased from BCRC (Hsin-Chu, Taiwan). B16F10 cells were cultured in DMEM supplemented with 10% FBS and penicillin-streptomycin (Logam, UT, USA) in a humidified atmosphere containing 5% CO_2 _at 37°C. Sample treatment was carried out 24 hrs after seeding.

### Tyrosinase activity assay

Tyrosinase activity was assessed as previously described [22]. Cells were plated in 6-well dishes at a density of 2 × 10^4 ^cells/well. B16 cells were incubated with different concentration of Cpc for 72 hrs, washed with ice-cold phosphate-buffered saline (PBS), centrifuged, and then treated with lysis buffer (phosphate buffer, pH 6.8, containing 1% Triton X-100, 0.1 mM PMSF, and 1 mM DTT). Cellular lysates were centrifuged at 12, 000 × *g *at 4°C for 15 min. The supernatants were collected, and the protein concentration was determined by Coomassie blue dye binding approach (Bio-Rad, Hercules, CA, USA). The extracted protein was stored at -80°C until use. The reaction mixture consisted of cell extract supernatant (30 μg) and 100 μL of L-DOPA (0.1%) in 0.1 M PBS (pH 7.0), and the tyrosinase activity was measured at 475 nm for 60 min. The reaction was carried out at 25°C.

### Melanin content determination

Melanin content was measured according to what was previously described, with slight modifications [23]. After co-culture with Cpc for 72 hrs, cells were washed twice with ice-cold PBS, centrifuged, and then treated with 1 N NaOH at 60°C for 10 min. The absorbances were measured sepctrophotometrically at 405 nm. Standard curves were derived from synthetic melanin (ranging from 0 to 200 μg/mL) in duplicate for each experiment. Melanin content was calculated by normalizing the total melanin values with protein content (μg of melanin/mg of protein) and expressed as a percentage of control. All the experiments were performed in triplicate on three independent occasions.

### Cytotoxicity analysis

The cell viability was determined by the 3-[4, 5-dimethylthiazol-2-yl]-2, 5-diphenyl tetrazolium bromide (MTT) assay as previously described [24]. MTT is a tetrazolium salt and is converted to insoluble formazan by mitochondrial dehydrogenase of living cells. Briefly, cells (5 × 10^4 ^cells/well) were seeded into 12-well plates. An aliquot of 50 μL MTT solution (1 mg/mL) was added to each well after removal of medium. The reaction was terminated after 4 hrs of incubation, and the resulted insoluble formazan was dissolved by further incubation with dimethyl sulfoxide (DMSO) for 10 min. The absorbance of each well at 570 nm was read for cell viability determination.

### cAMP content determination

Intracellular cAMP content was analyzed by a Direct cAMP enzyme immunoassay kit (Sigma-Aldrich, St. Louis, MO, USA) according to the manufacturer's instruction. Briefly, B16F10 cells were plated in 96-well dishes at a density of 5 × 10^4 ^cells/well. Cells were incubated with 0.1 mg/mL Cpc at different time intervals, and were lysed using 120 μL 0.1 N HCl for 10 min. Lysates were centrifuged at 600 × *g *at 25°C, and the supernatant was used directly.

### Immunoblotting

Cell lysates were run on a 10 or 15% SDS-PAGE gel and blotted onto nitrocellulose membranes. After blocking with 5% skin milk in TBST, proteins were identified using primary antibodies and HRP-conjugated secondary antibodies. The bands were visualized by ECL system (Amersham Pharmacea Biotech, U.S.). The antibodies used were: anti-β-actin (Temecula, CA, USA); anti-MITF (Calbiochem Darmstadt, Germany); anti-tyrosinase; anti-ERK (Franklin Lakes, NJ, USA); anti-pERK1/2; anti-MEK1/2; anti-p38; anti-p-p38; anti-CREB (Santa Cruz, CA, USA); anti-p-CREB (New England Biolabs, Beverly, MA); anti-c-phycocyanin (LTK BioLaboratories, Taipei, Taiwan).

### Total RNA extraction

Total RNA was extracted by TRIzol reagent (Invitrogen, Carlsbad, CA, USA). Cells were reacted with RNA extraction reagent for 5 min at room temperature, followed by an additional incubation for 3 min after the addition of chloroform (Merck, Darmstadt, Germany). The homogenates were centrifuged at 12000 × *g *for 15 min. RNA in aqueous phase were collected by isopropanol (TEDIA, Fairfield, CA, USA) precipitation, centrifuging at 12000 × *g *for 10 min, and stored in 75% ice-cold ethanol at -20°C until use.

### Quantitative PCR

Quantitative PCR (Q-PCR) was performed with reaction mixtures containing total RNA (100 ng), one-step RT-PCR Master Mix Reagents (Applied Biosystems, Foster City, CA, USA), and probes (*MITF, GAPDH*) on 7300 Real-Time PCR system (Applied Biosystems, Foster City, CA, USA).

### Reverse transcription-polymerase chain reaction (RT-PCR)

RT-PCR was performed by a two-step procedure, reverse transcription and PCR. Reverse transcription was carried out with a reaction mixture containing 1 μL oligo(dT)_18_, 5 μg total RNA, 1 μL 10 mM dNTP, and H_2_O at 65°C for 5 min. The reaction mixtures were then chilled on ice for 1 min, followed by the addition of 5 × first-strand buffer, 1 μL 0.1 M DTT and 1 μL Super Script™ III reverse transcriptase. The reaction mixtures were held at 50°C for 40 min, and then at 70°C for 15 min. The cDNA products were stored at 4°C. The PCR was carried out with the reaction mixtures containing 2 μL of cDNA product, 5 μL 10 × reaction buffer (Invitrogen, Carlsbad, CA, USA), 1 μL dNTP (MDBio, Taipei, Taiwan), 1.5 μL MgCl_2_, 1 μL Taq polymerase (MDBio, Taipei, Taiwan) and 1.25 μL of each forward (F) and reverse (R) primer. The primers included: Tyrosinase: F: 5'-GGCCAGCTTTCAGGCAGAG-GT-3', R: 5'-TGGTGCTTCATGGGCAAAATC-3'; GAPDH: F: 5'-GCACCACCAACTGCT-TAGC-3', R: 5'-TGCTCAGTGTAGCCCAGG-3'. PCR was performed with 30 cycles. Each cycle included denaturation at 94°C for 45s, primer annealing at 45°C for 45s, and primer extension at 72°C for 45s, and a final 10 min primer extension step at 72°C. The products were run on 10% agarose gels and stained with ethidium bromide.

### Immunofluorescence localization

Immunofluorescence localization was carried out as described previously [24]. Briefly, B16F10 cells were plated on glass cover slips and grown with or without Cpc. Cells were fixed with 2% paraformaldehyde in PBS for 20 min after three washes with PBS, followed by 0.1% Triton X-100/PBS for 3 min, and three washes. The coverslips were then incubated with blocking buffer (1% BSA) for 3 min, followed by three washes with PBS. Samples were immunostained with anti-Cpc-specific rabbit polyclonal antiserum (1:1000 dilution) in blocking buffer overnight at 4°C. The cells were washed with blocking buffer and incubated with FITC-conjugated goat anti-rabbit secondary antibodies (1:100 dilution) for 60 min. The coverslips were washed with PBS, treated with DAPI for 15 min, followed by further PBS washes. Confocal microscopy was performed with a Zeiss LSM700 microscope and images processed with Adobe Photoshop. Representative pictures were taken from three individual pictures.

### Statistical analysis

Data were presented as mean ± standard deviation. Statistical significance was analyzed by one-way ANOVA. Values of P < 0.05 were considered significant.

## Results

### Effects of Cpc on cell viability. tyrosinase activity, and melanin production

**Figure 1:**
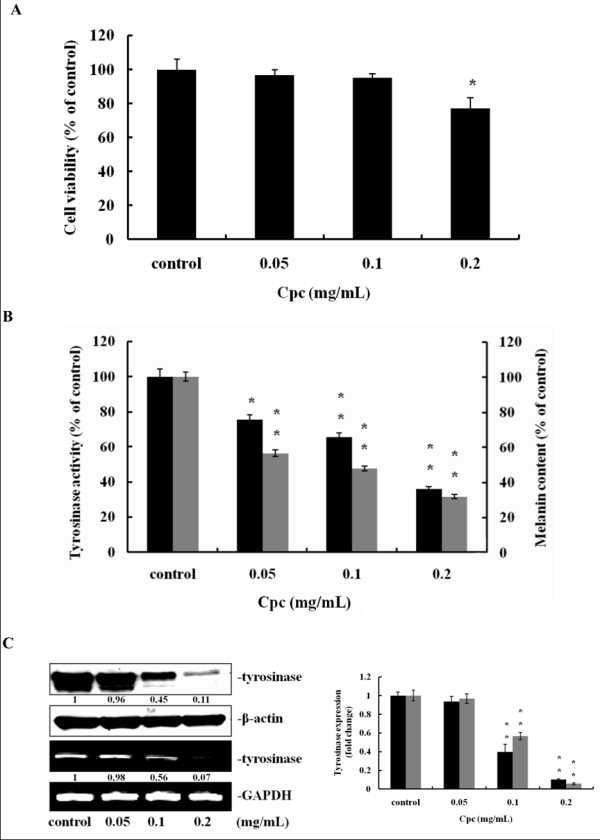
**Effect of Cpc on viability of B16F10 melanoma cell, tyrosinase activity and melanin contents**. Cells were treated with Cpc (0.05, 0.1, 0.2 mg/mL) for 72 hrs. (A) Cell viability was determined by MTT assay as described in Materials and Methods. (B) Tyrosinase activity (black) and melanin content (grey) were measured. (C) The expression of tyrosinase was determined by immunoblotting analysis (black) and RT-PCR (grey), using β-actin and GAPDH as internal standards, respectively. Data were expressed at mean ± SD from three different experiments. The asterisk (*) indicates a significant difference from control group (*, P < 0.05; **, P < 0.01).

Figure 1A shows the viability of B16F10 melanoma cells after treating with Cpc. The viability of melanoma cells was changed insignificantly at 0.05 and 0.1 mg/mL Cpc, except at a higher level of 0.2 mg/mL (77%). Based on the results of cell viability, the concentration of Cpc at 0.1 mg/mL was thus selected for the following study.

To investigate the antimelanogenic mechanism of Cpc, cellular tyrosinase activity and melanin content were measured. As indicated in Figure 1B, tyrosinase activity and melanin content were significantly (P < 0.05) and dose-dependently reduced from 75.7% to 65.7%, and 56.2% to 47.5%, respectively, with Cpc concentration ranging from 0.05 to 0.1 mg/mL. This suppression was further examined in the expression of tyrosinase at transcriptional and post-translational levels. As demonstrated in Figure 1C, Cpc significantly inhibited the expression of tyrosinase at both mRNA and protein levels, indicating that Cpc could modulate cellular machinery to attenuate melanogenesis in addition to Cpc's antioxidative property of reducing DOPAquinone back to DOPA.

### Effect of Cpc on α-MSH-stimulated Melanogenesis

**Figure 2:**
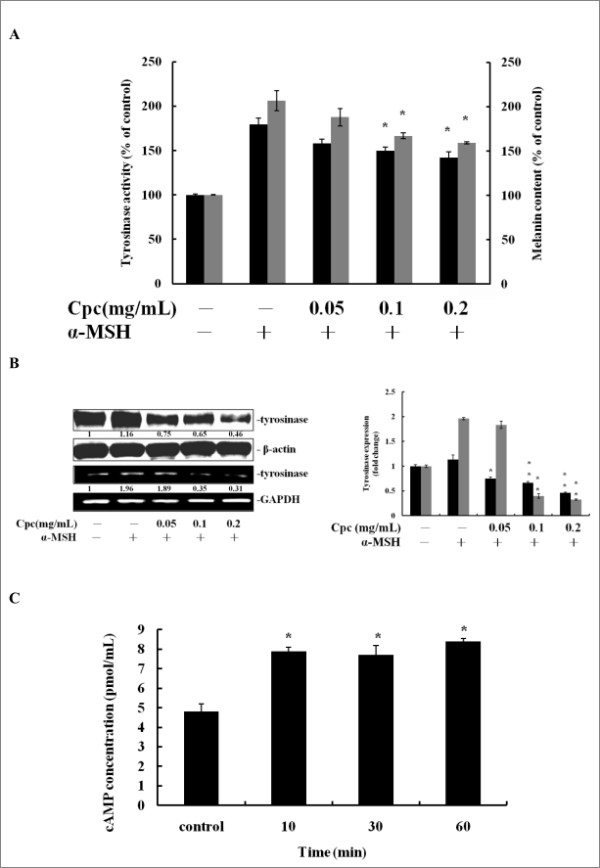
**Cpc attenuated α-MSH-stimulated melanogenesis and elevated the abundance of intracellular cAMP**. Cells were pretreated with 20 nM α-MSH for 30 mins, and then treated with Cpc (0.05, 0.1, 0.2 mg/mL) for 72 hrs. (A) Tyrosinase activity (black) and melanin content (grey) were measured. (B) The expression of tyrosinase was determined by immunoblotting analysis (black) and RT-PCR (grey), using β-actin and GAPDH as internal standards, respectively. (C) The cAMP concentration was measured by enzyme immunoassay at assigned time intervals (10, 30, 60 min) after Cpc treatment. Data were expressed at mean ± SD from three different experiments. The asterisk (*) indicates a significant difference from control group (*, P < 0.05).

Next, α-MSH, a cAMP elevating hormone facilitating melanocyte melanogenesis, was used to evaluate the potential mechanisms behind the Cpc-induced antimelanogenic effect. Figure 2A shows the changes of cellular tyrosinase activity and melanin content with the stimulation of α-MSH (20 nM). It was observed that the tyrosinase activity and melanin formation were inhibited in a dose-dependent manner with the increase of Cpc (0.05 to 0.1 mg/mL). Moreover, the expression of tyrosinase mRNA and protein was also suppressed by the treatment of Cpc (Figure 2B). Based on the above results, it was possible to suppose that Cpc could exert cAMP-associated signaling to regulate melaogenesis via manipulating α-MSH-induced melanogenesis. The cellular concentration of cAMP was then analyzed to further characterize the effect of Cpc. Figure 2C displays the cellular concentrations of cAMP measured 1 hr after Cpc treatment. The addition of Cpc (0.1 mg/mL) significantly enhanced the accumulation of cAMP from 4.8 to 7.9 pmol/mL at the first 10 min. These results might suggest linkage between cAMP and MAPK/ERK pathway [21] due to the decrease of tyrosinase gene expression and melanin synthesis. Thus, the activity of MAPK/ERK signaling pathway-associated molecules was further investigated.

### Effects of Cpc on the up-regulation of MAPK/ERK pathway and the down-regulation of MITF

**Figure 3:**
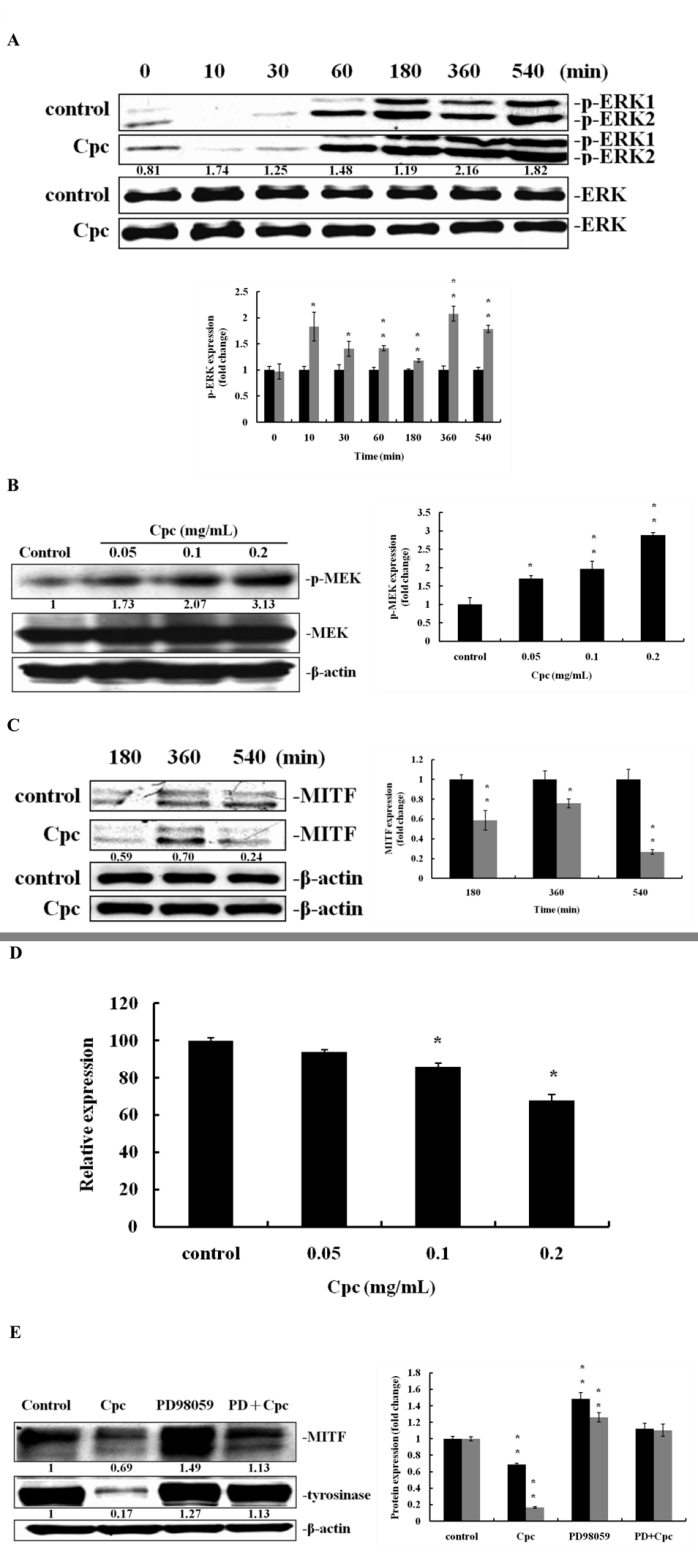
**Effect of Cpc on cAMP/MAPK/ERK pathway and MITF expression at protein and mRNA levels**. Immunoblot analysis was performed with cell extract proteins treated with (A) Cpc (0.1 mg/mL) at assigned time intervals for ERK1/2 (control (black); CPC-treated (grey)), and (B) different Cpc concentration (0.05, 0.1, 0.2 mg/mL) at 540 min for MEK. (C) Cell extract proteins at assigned time intervals treated with Cpc (0.1 mg/mL) were examined by Immunoblot analysis for MITF using β-actin as internal standards (control (black); CPC-treated (grey)). (D) Different levels of Cpc (0.05, 0.1, 0.2 mg/mL) treated *MITF *mRNA were analyzed by Q-PCR at 540 min. (E) Immunoblot analysis treated with Cpc (0.1 mg/mL), PD98059 (PD, 20 μM), and Cpc+PD at 72 hrs were performed for the evaluation of MITF and tyrosinase expression (MITF (black); tyrosinase (grey)). Data were expressed at mean ± SD from three different experiments. The asterisk (*) indicates a significant difference from control group (*, P < 0.05).

The Cpc-induced responses of MAPK/ERK pathway-associated factors, ERK 1/2 and MEK, were determined herein. Figure 3A shows the modulation of total ERK 1/2, and their phosphorylated counterparts, p-ERK1 and p-ERK2. The variation of total ERK1/2 was insignificant among groups. However, p-ERK1/2 significantly increased as early as 10 min after Cpc treatment. Moreover, the phosphorylation of MEK at 540 min was also significantly increased (Figure 3B). These results suggested that Cpc might activate the MAPK/ERK signaling.

As ERK-associated MITF degradation has been suggested [17], the level of MITF was thus investigated to characterize the antimelanogenic mechanism. Figure 3C displays the expression profile of MITF proteins after Cpc treatment. The expression of MITF protein was significantly inhibited at 540 min after Cpc (0.1 mg/mL) treatment. These results confirmed the findings that ERK critically modulates the Cpc-induced antimelanogenic effect. Moreover, the MITF mRNA level was investigated by Q-PCR to explore the upstream regulatory machinery. As seen in Figure 3D, the MITF mRNA levels decreased (P < 0.05) with the raise of Cpc indicating that Cpc likely influenced the activation of CREB, the transcription factor of MITF.

To further examine the involvement of MAPK/ERK pathway in Cpc-induced antimelanogenesis, an inhibitor of MEK, PD98059, was used to examine whether the Cpc-induced down-regulation of MITF and tyrosinase expression could be restored. As expected, the expression of MITF and tyrosinase was restituted with the treatment of PD98059 (Figure 3E). These results indicated that MAPK/ERK pathway plays an important role in the Cpc-induced antimelanogenesis in B16F10 melanoma cells.

### Down-regulatory effects of Cpc on p38 MAPK and CREB signaling

**Figure 4:**
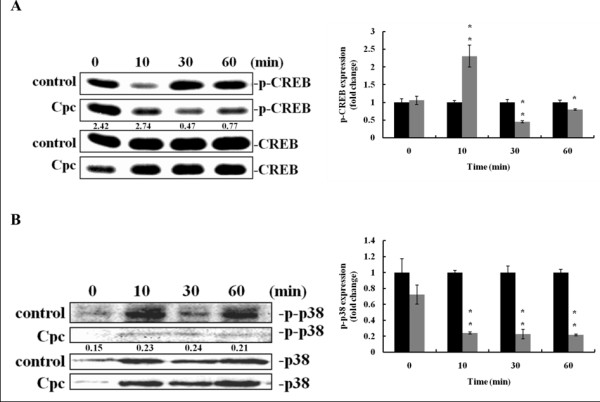
**The down-regulative effect of Cpc on p38 MAPK and CREB signaling pathways**. Cells were treated with Cpc (0.1 mg/mL). Immunoblot analysis was performed at assigned intervals for (A) CREB, and (B) p38 MAPK (control (black); CPC-treated (grey)).

Figure 4A depicts the down-regulatory effect of Cpc on the activation of CREB. The expression of p-CREB was markedly decreased at 30 min and 60 min after Cpc treatment, whereas no significant change was observed for the total CREB. These data indicated that CPC could hinder the phosphorylation of CREB leading to the subsequent reduction of MITF transcription, thereby restraining the following expression of tyrosinase. Furthermore, it is suggested that p38 MAPK can phosphorylate CREB to undergo nuclear translocation for gene transcription [25,26]. Our results showed that Cpc inhibited the phosphorylation of p38 (Figure 4B, at 10 min) leading to the decline of p-CREB.

### Cellular localization analysis

**Figure 5:**
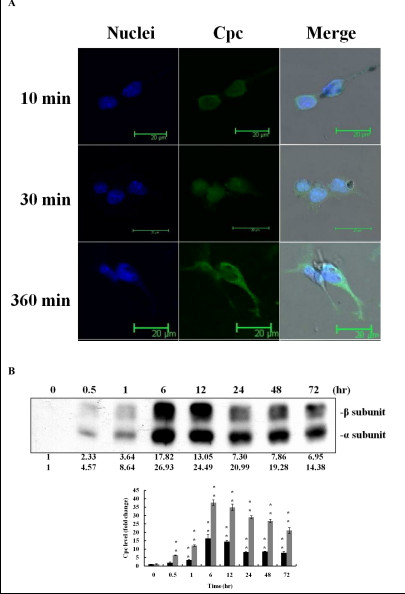
**The entry of Cpc into B16F10 melanoma cells**. Cells were treated with Cpc (0.1 mg/mL). (A) Confocal microscopy of Cpc localization at 6 hrs after treatment (1000 ×). (B) After washes with PBS, cells were lysed, and the extract proteins were analyzed by immunoblotting assay for Cpc at the assigned time intervals (β-subunit (black); α-subunit (grey)).

Cellular localization of Cpc was investigated by immunoblot analysis and confocal immunofluorescence localization study to explore the possible causes of the induced antimelanogenic effect on B16F10 melanoma cells. Confocal immunofluorescence localization study showed that Cpc entered into cells at 10 min, reached the nucleus at about 30 min after treatment, and then migrated to cytoplasm afterwards (Figure 5A). The subunits α/β of Cpc were clearly peaked at 6 and 12 hrs after administration (Figure 5B). These observations suggested that Cpc interacted with signal transduction molecules to potentiate the antimelanogenic effect.

## Discussion

In the present study, we demonstrated that Cpc is able to serve as a potential melanogenesis inhibitor. Our results suggested that Cpc inhibits melanin biosynthesis by dual mechanisms: the promoted degradation of MITF protein through the up-regulation of MAPK/ERK signaling pathway, and the suppressed activation of CREB via the down-regulation of p38 MAPK pathway. Cpc elevates the cellular abundance of cAMP, which triggers the activation of down-stream MAPK/ERK pathway, leading to the reduction of MITF proteins. It was reported that the activation of ERK1/2 resulted in the phosphorylation of MITF at S73, which induced the subsequent ubiquitin-dependent proteasomal degradation of MITF [17]. Moreover, the involvement of MAPK/ERK pathway was further confirmed by the treatment of MEK1/2 inhibitor, PD98059. On the other hand, Cpc may also exert its negative impact on p38 phosphorylation to restrict activation of the CREB, resulting in restricted *MITF *gene expression. A similar antimelanogenic effect was also described in that sulforaphane raised the level of p-ERK and reduced the abundance of p-p38 to inhibit the biosynthesis of melanin [27]. In addition, it is also suggested that Cpc could be used for treating ischemia-reperfusion injury through the activation of ERK pathway and suppression of p38 MAPK pathway [16].

The reciprocal steadiness between the activity of ERK and p38 is critical in governing melanogenesis [28,29]. As cAMP-elevating agents initiate the elevation of melanin synthesis, the antagonistic reactions for the decline of melanogenesis via the activation of MAPK pathway start to proceed. These retrocontrol mechanisms may be designed to guard the steady-state of melanin synthesis. It is also indicated that the treatment of a pyridinyl imidazole cell-permeable p38 inhibitor, SB203580, was able to increase phosphorylation of ERK [28], whereas inactivation of MEK1/2 could stimulate α-MSH-induced p38 MAPK activity [30]. Accordingly, the external stress signals such as heat shock, ultraviolet light, irradiation, osmotic stress, and proinflammatory cytokines, -induced melanin pigment formation via p38 MAP kinase signaling can be regulated. In agreement with these findings, Cpc might also exert similar reciprocal mechanism to down-regulate the synthesis of melanin.

Several signal transduction pathways have been revealed to balance melanin pigment formation. These pathways have been suggested to converge on CREB [31] to facilitate the expression of melanogenesis-associated proteins. The p38 MAPK pathway has been implied to pass the stimuli after the burst phase of cAMP/PKA signaling [32]. Once the p38 MAPK signaling is disturbed, this will cause either the impediment or detour of the stimuli, consequently leading to suppression of the activation of CREB. Consequently, the expression of melanogenic enzymes (tyrosinase, TRP-1, DCT) is hampered due to the limited expression level of MITF. In our study, Cpc was found to inhibit the activation of p38 MAPK, thereby attenuating melanin synthesis.

Finally, the structure resemblance of Cpc constituents to MAPK pathway modulators, for example SB203580 and bilirubin, could possibly in part account for its antimelanogenic effect. SB203580 [4-(4'-fluorophenyl)-2-(4'-methylsulfinylphenyl)-5-(4'-pyridyl) imidazole] acts as a competitive inhibitor of ATP binding of MAP kinase homologues p38α, p38β and p38β2, and blocks α-MSH-induced melanogenesis in B16 cells [33]. It is likely that phycocyanobilin, the prosthetic group of Cpc, might possess similar pyridinyl imidazole structural features to that of SB203580, sharing comparable inhibitory mechanisms. In constrast, a tetrapyrrole structurally related molecule of phycocyanobilin, bilirubin, was demonstrated to have an antitumoral activity through the activation of MAPK/ERK pathway [34]. This activity might be a clue for us to explore the details of Cpc-induced MITF degradation through MAPK/ERK pathway.

The existence of Cpc in melanoma cells was evidenced by the analyses of immunoblotting and confocal immunofluorescence localization. Cpc was found to be at nucleus at the early stage (10 and 30 min) of entrance and then accumulated at cytoplasm afterwards (360 min). These observations might infer that the constituents of Cpc, such as phycocyaniobilin, could function as either or both a p38 MAP kinase inhibitor and an ERK activator to regulate melanin synthesis. Further in-depth studies will be conducted to justify this assumption.

## Conclusions

**Figure 6:**
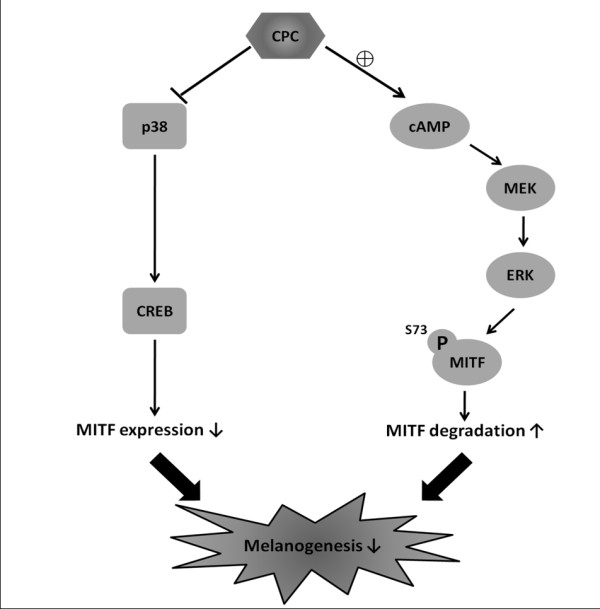
**The scheme of Cpc-induced antimelanogenic effect on B16F10 melanoma cells**. A schematic representation of the actions of Cpc with respect to associated signaling pathways in B16F10 cells.

Cpc effectively restrained the expression of tyrosinase, the rate-limiting enzyme of melanogenesis, through the regulatory mechanisms at transcriptional (through p38 MAPK pathway on CREB activation) and post-translational (through MAPK/ERK pathway on MITF phosphorylation/degradation) levels. This phycobiliprotein exerted combinatory activities including antioxidative capacity and the regulative ability of tyrosinase expression (Figure 6) to modulate melanogenesis. Its applications could be applied widely in food, cosmeticeutical, and biomedical industries.

## Competing interests

The authors declare that they have no competing interests.

## Authors' contributions

LCW conceived the study, and participated in the experiment design and project coordination. He was also responsible for drafting the manuscript. YYL carried out the determination of tyrosinase activity and melanin content. She also performed the RTPCR, QPCR, and immunoblot analyses. SYY conducted the immunofluorescence localization and immunoblot analysis. YTW and YTT determined the cAMP content and performed immunoblot analyses. All authors read and approved the final manuscript.
